# Diphtheria

**Published:** 1879-06-20

**Authors:** W. W. Carpenter

**Affiliations:** California


### *DIPHTHERIA.*

BY W. W. CARPENTER, M.D., OF CALIFORNIA.

EDITOR *Southern Medical Record :*

In the February number of your excellent journal chlorate potass is recommended as the best remedy yet used in diphtheria. I deprecate such teaching. There is nothing in etiology more certainly established than that diphtheria is cryptogamic in origin.

Such being the fact, the remedy should be anti-cryptogamic. Such a remedy, in its greatest power and perfection, is the liquefied gas acid sulphurosii. Potassa chloras, ferri mur. and their class constitute the chlorine treatment—a treatment based on the chemical theory of contagium. As a remedy in diphtheria it addresses itself to the *effect* without any regard to the *cause* of the disease. If it oxygenates the blood faster than the bacteria devitalizes it by abstracting that vital element therefrom, you may eventually save your patient. But even in that event it will take you fifteen days to accomplish what you would effect in twelve hours with acid sulphurous.

But potassa chloral cannot be borne in sufficient quantity to cure an asthenic case of diphtheria without causing a dangerous, if not fatal, depression of the heart. If combined with quinine or any of its alkaloids to steady the heart's action, it can be safely borne in larger doses. In fact, the physician who would exhibit it alone is to be pitied. In union with tinct. ferri mur. and quinia, we not only have a much more effective remedy, but a far safer one.

℞ Acid sulphurosii.......................................... ʒ vi

Glycerine .............................................. ℥ iv

Sol. potassa chloras, ad ................................. ℥ viii

Sig. From ½ teaspoonful to two teaspoonfuls every half hour, according to age. Small and frequent doses give us all the topical application required.

